# Changes in COVID‐19 measures in the workplace: 8‐month follow‐up in a cohort study of full‐time employees in Japan

**DOI:** 10.1002/1348-9585.12273

**Published:** 2021-09-14

**Authors:** Hiroki Asaoka, Natsu Sasaki, Kotaro Imamura, Reiko Kuroda, Kanami Tsuno, Norito Kawakami

**Affiliations:** ^1^ Department of Psychiatric Nursing Graduate School of Medicine The University of Tokyo Tokyo Japan; ^2^ Department of Mental Health Graduate School of Medicine The University of Tokyo Tokyo Japan; ^3^ Division for Environment, Health and Safety The University of Tokyo Tokyo Japan; ^4^ School of Health Innovation Kanagawa University of Human Services Kanagawa Japan

**Keywords:** COVID‐19, employees, infection control measures, workplace

## Abstract

**Objectives:**

It is unclear how many workplace COVID‐19 preventive measures were maintained during repeated outbreaks. The aim of this study was to investigate a longitudinal change of implementation of workplace preventive measures responding to COVID‐19 in Japan.

**Methods:**

An online longitudinal study was conducted using a cohort of full‐time employees in Japan, starting in March 2020 (T1), with follow‐up surveys in May (T2), August (T3), and November (T4) 2020. A repeated measures analysis of variance was performed to compare the difference among the four surveys in the mean number of 23 predetermined items of the measures implemented.

**Results:**

The final sample comprised 800 employees. The mean number of the implemented measures increased from T1 to T2, but did not change from T2 to T3, then decreased from T3 to T4. The number of workplace preventive measures significantly increased from T1 to T2 for 21 items (*P* < .001), and significantly decreased from T3 to T4 for 14 items (*P* < .001 to *P* = .005).

**Conclusions:**

While the preventive measures responding to COVID‐19 in the workplace were well‐implemented during the earlier phase of the outbreak, they seem to have been relaxed after a huge outbreak (T3 to T4: August to November 2020). Workplaces should be encouraged to continue the preventive measures over repeated outbreaks.

## 1 INTRODUCTION

**FIGURE 1:**
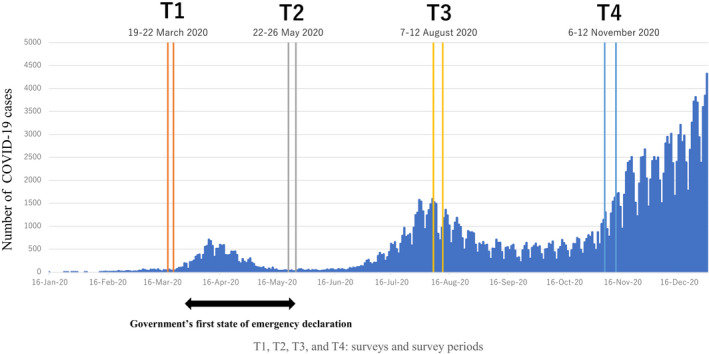
Trends of daily new confirmed COVID‐19 cases in Japan and each survey timing

The transmission of the novel coronavirus disease (COVID‐19) has been spreading in Japan; the number of confirmed cases and deaths due to COVID‐19 had increased to 472 112 and 9113, respectively, as of 31 March 2021 (Trends of Daily new confirmed COVID‐19 cases in Japan shown in Figure 1).^1^ The declaration of the first state of emergency in April 2020 helped the Japanese government control the pandemic's trajectory in Japan,^2^ and the state of emergency was lifted in late May 2020. Although several countries imposed strict lockdown measures to curb the spread of the disease, the measures in the declaration of a state of emergency in Japan lacked legal authority and depended on citizens’ self‐restraint. The Japan Society for Occupational Health, in conjunction with the Japanese Society of Travel Medicine, published guidelines for preventive measures of COVID‐19 in the workplace in June 2020 and made an addendum to the guideline on 11 August 2020: medium to long‐term measures after business resumption (ie behavioural change including education for employees, environmental optimization, workstyle reform), prevention for workplace bullying and harassment related to COVID‐19.^3^ The number of COVID‐19 cases in Japan increased rapidly from the beginning of July to the beginning of August after the first emergency declaration was lifted, but it decreased after 7 August and did not increase rapidly again until late November.^1^ During this period, the Japanese government implemented a policy to resume economic activities, such as encouraging the population to eat at restaurants with its “Go to eat” campaign and engage in domestic travel through its “Go to travel” campaign.^4^ On 7 January 2021, Japan has declared its second state of emergency regarding COVID‐19 in the capital, Tokyo, and three surrounding prefectures, as the number of daily infections surged and hospitals in Tokyo reported nearly 80% occupancy.^5^

Non‐pharmaceutical interventions in the workplace, such as disinfection of the work environment and encouraging telework and telecommuting were reported as important preventive measures of COVID‐19 and were expected to be implemented immediately in the pandemic.^6^, ^7^ During the 2009 influenza A (H1N1) pandemic, a randomized controlled trial reported that the adoption of combined workplace measures, including the measurements of body temperature each day and the obligation for symptomatic workers to stay at home, reduced the overall risk for infection transmission by 20% in the workplace.^8^ The guidelines of the United States Centers for Disease Control (CDC) suggested similar evidence‐based appropriate workplace measures for COVID‐19: conducting daily health checks up, encouraging of wearing masks, social distancing in the workplace.^9^ In an early phase of the pandemic in Japan, around the first state of emergency in April and May, our previous cross‐sectional study revealed that about 80% of employees were under some workplace preventive measure.^10^ The proportion increased in May 2020, showing that most workplaces made an effort to establish preventive measures to respond to COVID‐19 between March 2020 and May 2020.^11^

World Health Organization (WHO) reported an increasing attitude of apathy or resistance towards adherence to major non‐pharmaceutical interventions as an expected and natural reaction to the prolonged nature of this crisis and the associated inconvenience and hardship, termed “pandemic fatigue”; there also was concern about the decline of workplace measures for COVID‐19.^12^ For instance, A report of US residents showed a decrease in reported adherence to non‐pharmaceutical interventions during the pandemic between April and November 2020.^13^ Workplace measures also were assumed to decrease during the pandemic, however, no long‐term cohort study has been reported and the trend in the rate of implementation of workplace measures has not been quantified. Workplace measures relating to COVID‐19 are important not only to prevent infection in the workplace, but also to prevent the spread of infection in the community.^6^ It is important to know how sustainable the workplace measures are that were implemented to prevent the transmission of COVID‐19 in the workplace during repeated outbreaks, following our previous studies in the early phase of the outbreak.^10^, ^11^, ^14^

The aim of this study was to investigate the longitudinal change of implementation of preventive measures responding to COVID‐19 in the workplace in Japan over repeated outbreaks, extending the follow‐up to August and November 2020 when the second (in late July and August 2020) and third outbreaks (November 2020+) occurred.

## 2 METHODS

### 2.1 Study design

The cohort was established from the panel of an Internet survey company, and included full‐time employees aged 20‐59 years old, living in Japan in February 2019. The sample was retrieved with an equal number of participants in each of eight cells stratified by gender and age (20‐29, 30‐39, 40‐49, and 50‐59). The cohort was composed of 4120 employees. The longitudinal analysis was conducted within that cohort, followed by online surveys.^10^, ^11^ The cohorts were invited to participate in the baseline survey of this study online on March 19‐22, 2020 (T1). The respondents in T1, after excluding the unemployed, were invited to participate on May 22‐26, 2020 (T2), about 1.5 months after the Japanese Cabinet office declared a state of emergency in response to COVID‐19.^2^ The respondents in T2, after excluding the unemployed, were invited to participate on August 7‐12 2020 (T3). The respondents in T3 were invited to participate in the follow‐up survey on 6‐12 November 2020 (T4).

This study protocol was approved by the research ethics committee of the graduate school of medicine/faculty of medicine, University of Tokyo (no. 10856‐(2)(3)(4)(5)). The study conformed to the strengthening the reporting of observational studies in epidemiology (STROBE) guidelines.^15^

### 2.2 Participants recruitment

**FIGURE 2:**
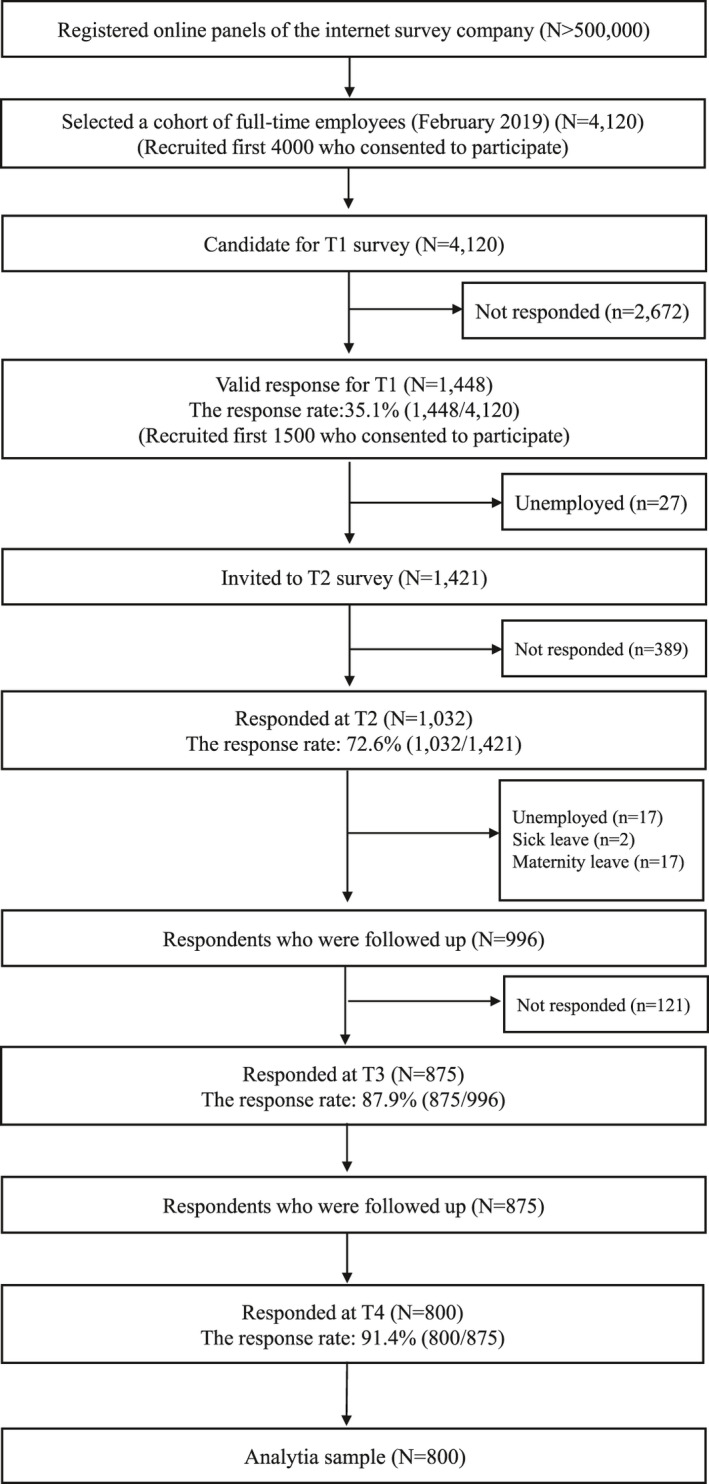
Flowchart of participant recruitment

Participants of the study were a sample of full‐time employees living in Japan, recruited from a database of over 500,000 workers created by an Internet survey company. We planned to recruit 1500 participants from 4120 respondents who participated in a previous survey in 2019 conducted by the Internet survey company.^16^ A total of 1448 (35.1%) participants completed an online questionnaire at T1 (March 2020). After excluding respondents who were unemployed (n = 27) at T1, we followed the remaining 1421 respondents and surveyed them at T2 (May 2020), T3 (August 2020), and T4 (November 2020). A total of 1032 (72.6%) participants completed the follow‐up questionnaire at T2, excluding 389 non‐respondents; we further excluded respondents who were unemployed (n = 17), on sick leave (n = 2), and on maternity leave (n = 17) at T2. Among the remaining 996 respondents who were followed further, 875 (87.9%) participants responded at T3, with 121 non‐respondents; among them, 800 (91.4%) responded at T4, with 75 non‐respondents (Figure 2). Reasons of non‐responses were not assessed. We limited the analyses to data of these 800 respondents who completed all four surveys (follow‐up rate to the initial 1441 employed respondents, 55.5%). Participants received a small token as a reward. The participants of the study were fully informed the aim, procedure, and ethical and privacy considerations, and informed consent was obtained.

### 2.3 Measurements

#### 2.3.1 Workplace measures to respond to COVID‐19: preventive workplace measures

The list of measures taken in the workplace to respond to COVID‐19 was originally developed based on previous literature of the novel influenza (H1N1),^17^ through discussion among occupational physicians (NS, RK, and NK) who engaged in workplace COVID‐19 prevention in Japan. The 23 items were categorized: (a) prevention to be taken by individuals; (b) prevention to reduce the risk of infection in the workplace; (c) criteria and procedures for waiting at home and for clinical contact; (d) temporary leave when infected or in a pandemic; (e) information about consideration for high‐risk people; (f) introduction of reliable information resources; and (g) information on the duration of special measures. The response was dichotomized: Yes (“already implemented during normal times” and “newly implemented” on T1, “already implemented before the previous survey” and “newly implemented after the previous survey” on T2, T3, and T4) or No (“not implemented,” and “not applicable for my work.”). We calculated the number of preventive measures among 23 items in T1, T2, T3, and T4.

#### 2.3.2 Demographic variables

As socio‐demographic variables, gender, age, marital status, the number of children, educational attainment, type of industry, company size, occupational type, and geographic block were retrieved at T1 survey. The type of industry was classified into 14 groups according to Japan Standard Industrial Classification by Ministry of Internal Affairs and Communications.^18^ Company size was categorized into ≥1000, 300‐999, 50‐299, and <50 employees. The occupational type was classified: managers, non‐manual, and manual workers. The living area was identified using the standard classification of geographic block in Japan (Hokkaido, Tohoku, Kanto, Chubu, Kansai [Kinki], Chugoku & Shikoku, Kyushu & Okinawa).

### 2.4 Statistical analysis

Descriptive analysis was conducted to examine the change of the frequency in workplace measures of 23 items responding to COVID‐19 at T1, T2, T3, and T4. A repeated measure analysis of variance (ANOVA) was performed to compare the difference in the mean numbers of the 23 implemented measures during each of the four stages of the surveys (the Bonferroni method was used for post‐hoc analysis). The Cochran's *Q* test and the McNemar test were used to assess differences in the proportion of implemented measures between T1, T2, T3, and T4. Statistical significance for the Cochran's *Q* test was set as a two‐sided *P* < .05. A statistical significance for the repeated measures ANOVA and the McNemar test was set as a two‐sided *P* < .008 (=0.05/6), depending on the number of multiple tests (n = 6), to prevent an inflation of the type 1 error. All analyses were conducted using SPSS version 26.0J for Windows (SPSS).

## 3 RESULTS

The final sample comprised of 800 employees. The characteristics of participants are shown in Table 1. The mean age was 41.9 years old (SD = 10.3; range: 22‐60). Most participants were non‐manual workers (63.1%), in the large company size (≧1000 employees, 32.4%), in the manufacturing industry (25.3%), and living in Kanto geographic block (41.3%).

**TABLE 1:** Participants’ characteristics for full‐time employees in Japan (N = 800)

	N (%)	Mean (SD) [min‐max]
Gender		
Male	424 (53.0)	
Female	376 (47.0)	
Age		
20‐29 years old	127 (15.9)	41.93 (10.30) [22 ‐ 60]
30‐39 years old	216 (27.0)	
40‐49 years old	226 (28.2)	
50‐59 years old	218 (27.3)	
≥60 years old	13 (1.6)	
Marital status		
Unmarried	401 (50.1)	
Married	399 (49.9)	
Presence of children		
Childless	463 (57.9)	
With children	337 (42.1)	
Educational attainment^a^		
Junior high school	6 (0.8)	
High school	180 (22.5)	
Junior college/vocational school/technical college	185 (23.1)	
University	384 (48.0)	
Graduate school	45 (5.6)	
Type of industry		
Manufacturing	202 (25.3)	
Medical and welfare	103 (12.9)	
Retail and wholesale business	77 (9.6)	
Finance, insurance, real estate	66 (8.3)	
Public service	65 (8.1)	
Information and technology services	67 (8.4)	
Life‐related services and entertainment	54 (6.8)	
Professional and technical services	43 (5.4)	
Transportation	12 (4.5)	
Education and learning support	36 (4.5)	
Construction	29 (3.6)	
Eating/drinking, hotel business	12 (1.5)	
Agriculture and industry	3 (0.4)	
Others	7 (0.9)	
Company size		
≧1000 employees	259 (32.4)	
300‐999	144 (18.0)	
50‐299	213 (26.6)	
<50	161 (20.1)	
Unknown	23 (2.9)	
Occupational type		
Managers	92 (11.5)	
Non‐manual	505 (63.1)	
Manual	203 (25.4)	
Geographic block		
Hokkaido	32 (4.0)	
Tohoku	43 (5.4)	
Kanto	330 (41.3)	
Chubu	146 (18.3)	
Kansai (Kinki)	136 (17.0)	
Chugoku & Shikoku	61 (7.6)	
Kyushu & Okinawa	52 (6.5)	

Abbreviation: SD, standard deviation.

^a^ The education attainment was measured at T2.

The mean number of implemented measures among 23 items are compared at T1, T2, T3, and T4 in Table 2. The repeated measures ANOVA showed a significant difference in the mean number of implemented measures at each time point of the survey (*F* = 193.31, *P* < .001). As a result of the multiple comparison procedure, the mean number of implemented measures at T2 significantly increased from T1 (+3.4, *P* < .001); at T3 significantly increased from T1 (+3.6, *P* < .001) but was not significantly different from T2; at T4 significantly increased from T1 (+2.1, *P* < .001) but significantly decreased from T2 (−1.3, *P* < .001) and T3 (−1.5, *P* < .001).

**TABLE 2:** The crude means and test for difference between means of the number of preventive measures among 23 items at baseline (T1), T2, T3, and T4 during the COVID‐19 pandemic among the cohort of Japanese employees (N = 800)

Survey (time of survey)	The number of preventive measures among 23 items		Test for difference between means of two time points		
	Mean	SD	T2	T3	T4
T1 (March 2020)	11.3	6.0	−3.4**	−3.6**	−2.1**
T2 (May 2020)	14.6	5.7	–	−0.3	1.3**
T3 (August 2020)	14.9	5.9	–	–	1.5**
T4 (November 2020)	13.4	6.2	–	–	–
Test for difference	*F* = 193.31, *P* < .001				

Note

COVID‐19: Coronavirus disease 2019; SD: standard deviation.

** *P* <.001.

The frequencies of implemented measures of the 23 items for COVID‐19 at T1, T2, T3, and T4 are shown in Table 3. The Cochran's *Q* test showed a significant difference in the implementation rate of each of the 23 items at T1, T2, T3, and T4 (*P* < .001), and implementation of any of the 23 preventive measures at T1, T2, T3, and T4 (*P* < .001). The implementation rate of most workplace preventive measures significantly increased from T1 to T2. From T2 to T3, the implementation rates of three items of most workplace preventive measures significantly changed (enforcement of temperature measurement, changing the working environment, and announcement of reliable information collection destinations) (*P* < .001 to *P* = .003), while the other items did not. From T3 to T4, the implementation rate of 14 of the 23 items significantly decreased (*P* < .001 to *P* = .005). Comparing the implemented measures of 23 items at T1 and T4, 18 items significantly increased. Implementation of any of the 23 preventive measures at T2 (99.0%, *P* < .001), T3 (99.3%, *P* < .001) significantly increased from T1 (96.6%).

**TABLE 3:** The change in frequency of implementation of preventive measures for COVID‐19 by companies, reported by Japanese workers (N = 800)

	T1 19‐22 March 2020		T2 22‐26 May 2020		T3 7‐12 August 2020		T4 6‐12 November 2020		P for Cochran's *Q* test	P for difference (McNemar)					
	n	%	n	%	n	%	n	%	T1, T2, T3, T4	T1‐T2	T1‐T3	T1‐T4	T2‐T3	T2‐T4	T3‐T4
(a) Prevention taken by individuals															
Hand washing, gargle enforcement	702	87.8	708	88.5	726	90.8	740	92.5	<0.001^††^	0.624	0.025	<0.001^**^	0.086	0.002^*^	0.166
Encourage finger alcohol disinfection	695	86.9	741	92.6	758	94.8	737	92.1	<0.001^††^	<0.001^**^	<0.001^**^	<0.001^**^	0.061	0.746	0.016
Encourage wearing masks	639	79.9	758	94.8	767	95.9	749	93.6	<0.001^††^	<0.001^**^	<0.001^**^	<0.001^**^	0.243	0.328	0.026
Enforce cough etiquette	632	79.0	674	84.3	686	85.8	714	89.3	<0.001^††^	0.001^*^	<0.001^**^	<0.001^**^	0.303	<0.001^**^	0.010
Enforcement of temperature measurement	394	49.3	583	72.9	634	79.3	576	72.0	<0.001^††^	<0.001^**^	<0.001^**^	<0.001^**^	<0.001^**^	0.664	<0.001^**^
(b) Prevention to reduce the risk of infection at workplace															
Cancel or postpone internal or external business events	488	61.0	556	69.5	562	70.3	497	62.1	<0.001^††^	<0.001^**^	<0.001^**^	0.609	0.681	<0.001^**^	<0.001^**^
Disinfection of the work environment	309	38.6	530	66.3	558	69.8	523	65.4	<0.001^††^	<0.001^**^	<0.001^**^	<0.001^**^	0.041	0.704	0.027
Refrain from traveling overseas	286	35.8	315	39.4	315	39.4	334	41.8	0.002^††^	0.017	0.023	0.001^*^	1.000	0.171	0.153
Restrictions on eating, drinking, and entertainment for work	269	33.6	419	52.4	434	54.3	339	42.4	<0.001^††^	<0.001^**^	<0.001^**^	<0.001^**^	0.316	<0.001^**^	<0.001^**^
Enforcement of staggered work	274	34.3	378	47.3	385	48.1	249	31.1	<0.001^††^	<0.001^**^	<0.001^**^	0.63	0.554	<0.001^**^	<0.001^**^
Encourage telework and telecommuting (including remote work)	225	28.1	430	53.8	410	51.3	287	35.9	<0.001^††^	<0.001^**^	<0.001^**^	<0.001^**^	0.055	<0.001^**^	<0.001^**^
Changing the working environment (desk layout, flow lines, installing vinyl curtains, etc)	136	17.0	360	45.0	450	56.3	336	42.0	<0.001^††^	<0.001^**^	<0.001^**^	<0.001^**^	<0.001^**^	0.177	<0.001^**^
Restrictions on the use of employee cafeterias	116	14.5	281	35.1	268	33.5	236	29.5	<0.001^††^	<0.001^**^	<0.001^**^	<0.001^**^	0.255	0.001^*^	0.005^*^
(c) Criteria and procedure for waiting at home and clinical contact															
Request to refrain from going to work when ill	618	77.3	679	84.9	677	84.6	586	73.3	<0.001^††^	<0.001^**^	<0.001^**^	0.025	0.923	<0.001^**^	<0.001^**^
Report request for fever	566	70.8	651	81.4	653	81.6	614	76.8	<0.001^††^	<0.001^**^	<0.001^**^	0.001^*^	0.924	0.004^*^	0.001^*^
Dissemination of information on home remedies and consultations for COVID‐19	494	61.8	602	75.3	575	71.9	498	62.3	<0.001^††^	<0.001^**^	<0.001^**^	0.842	0.034	<0.001^**^	<0.001^**^
Waiting at home if you have a history^a^ of staying abroad	207	25.9	304	38.0	288	36.0	308	38.5	<0.001^††^	<0.001^**^	<0.001^**^	<0.001^**^	0.253	0.833	0.157
(d) Temporary leave when infected or pandemic															
Providing information on how to deal with infected cases in the workplace	466	58.3	584	73.0	580	72.5	468	58.5	<0.001^††^	<0.001^**^	<0.001^**^	0.950	0.818	<0.001^**^	<0.001^**^
Providing information on compensation when waiting at home	286	35.8	434	54.3	442	55.3	379	47.4	<0.001^††^	<0.001^**^	<0.001^**^	<0.001^**^	0.619	0.001^**^	<0.001^**^
Provision of information on compensation when taking leave due to infection	273	34.1	412	51.5	412	51.5	381	47.6	<0.001^††^	<0.001^**^	<0.001^**^	<0.001^**^	1.000	0.057	0.048
(e) Information about consideration for high‐risk people															
Consideration for staff who are at high risk of serious illness in case of infection (elderly people, pregnant women, etc.)	323	40.4	460	57.5	449	56.1	391	48.9	<0.001^††^	<0.001^**^	<0.001^**^	<0.001^**^	0.505	<0.001^**^	<0.001^**^
(f) Introduction of reliable information resources															
Announcement of reliable information collection destinations (such as the Ministry of Health, Labor and Welfare website)	345	43.1	424	53.0	467	58.4	416	52.0	<0.001^††^	<0.001^**^	<0.001^**^	<0.001^**^	0.003^*^	0.655	0.001^*^
(g) Information on the duration of special Measures															
Providing information on how long special measures will be taken	272	34.0	433	54.1	396	49.5	357	44.6	<0.001^††^	<0.001^**^	<0.001^**^	<0.001^**^	0.023	<0.001^**^	0.014
Implementation of any of the 23 preventive measures	773	96.6	792	99.0	794	99.3	786	98.3	<0.001^††^	<0.001^**^	<0.001^**^	0.037	0.727	0.210	0.057

Abbreviation: SD, standard deviation.

COVID‐19: Coronavirus disease 2019.

^a^ The item was not restricted to any countries or any period about the history of staying abroad.

^†^ *P* <.05, ^††^ *P* <.01 for Cochran's *Q* test. *P <.008, **P <.001 for McNemar test.

## 4 DISCUSSION

The mean number of implemented measures among the 23 items for COVID‐19 in the Japanese workplaces increased from T1 (March 2020) to T2 (May 2020), and did not change from T2 to T3 (August 2020), however, it decreased from T3 to T4 (November 2020). The implementation rates of most workplace preventive measures for COVID‐19 significantly increased from March 2020 to May 2020. Most of the measures were implemented at a rate greater than 50% in May 2020 and August 2020. From August 2020 to November 2020, the implementation rate of 14 of the 23 items significantly decreased (*P* < .001 to *P* = .005). The results indicate that the preventive measures responding to COVID‐19 in the workplace were well‐implemented during the earlier phase of the outbreak, however, these may have been relaxed from August to November 2020.

This study showed that the mean number of implemented measures for COVID‐19 in the workplace in Japan did not significantly change from May 2020 to August 2020, although it increased from March 2020 to May 2020, as shown in our previous studies.^10^, ^11^ With the declaration of the first state of emergency, companies were comprehensively promoting the implementation of workplace preventive measures during April and May 2020. When the emergency state ended, the Japanese government called on business associations to step up efforts to prevent infections in the workplace and carefully monitor the health conditions of workers.^19^ This might have encouraged companies to continue to implement preventive measures for COVID‐19 in the workplace, and maintain them from May 2020 to August 2020. Relating to the implementation rates of workplace preventive measures, most did not significantly change from May to August 2020. However, significant increases were observed in enforcement of temperature measurement, changing the working environment, and announcement of reliable information collection destinations (*P* < .001 to *P* = .003). The implementation rates of most workplace preventive measures were maintained even after the declaration of the first state of emergency ended. This may be because the guidelines of CDC and the Japan Society for Occupational Health were revised,^3^, ^9^ since they had promoted the daily health checkup of employees and disinfection of the work environment.

On the other hand, the implementation of preventive measures decreased from August to November 2020. There are several possible reasons for the decline in the implementation of preventive measures from August to November 2020. The first reason may be the change in policy by the Japanese government. The Prime Minister announced the resumption of economic activities on July 22,^4^ such as encouraging the population to eat in restaurants with its “Go to eat” campaign and engage in domestic travel through its “Go to travel” campaign. The number of users of Go to Travel was higher in October and November than in August 2020,^20^ and the number of reported users was higher in the T4 survey than in the T3 survey. The government's promotion of economic activities might have made companies relax the relevant implementation of preventive measures, for example, restrictions on eating, drinking, and entertainment for work, from August to November 2020. In addition, some companies resumed office commuting in accordance with the government's policy of resuming economic activity, and these companies may have relaxed the implementation rate of staggered work, telework, and telecommuting.

The second reason may be the lack of rapid change in the trend of the number of people infected with COVID‐19 between August and November 2020. With no rapid increase in the number of COVID‐19 cases and the number of cases remaining low between August and November 2020,^1^ companies may have relaxed the implementation of various preventive measures in their workplaces.

The third possible reason is the fact that companies were not obliged but requested to implement preventive measures in their workplaces after the first declaration of the state of emergency was lifted^19^; thus, the implementation of workplace preventive measures was left to the discretion of each company.

As a fourth reason, the prolonged COVID‐19 pandemic had led to a decline in the implementation of preventive measures in the workplace. Japanese companies had been required by the Japanese government to implement preventive measures for COVID‐19 infection in their workplace since early April 2020. WHO reported that the increasing attitude of apathy or resistance towards adherence to major non‐pharmaceutical interventions as an expected and natural reaction to the prolonged nature of this crisis and the associated inconvenience and hardship, and there is also concern about the decline of workplace measures of COVID‐19.^12^ In addition, a previous study among US residents found a decrease in reported adherence to non‐pharmaceutical interventions overall and to most individual non‐pharmaceutical interventions during the COVID‐19 pandemic between April and November 2020.^13^ As shown in these reports, the implementation of preventive measures in the workplace in Japanese companies may have been relaxed between August and November 2020 with the prolongation of the pandemic. In addition, the prolonged duration of the pandemic may have made it economically difficult for companies to implement of preventive measures in their workplaces, which may have reduced the implementation of various items of preventive measures in their workplaces.

From August to November 2020, there was a decrease in the implementation rate of several items that are particularly important for infection control of COVID‐19^3^, ^9^, ^21^, ^22^: enforcement of temperature measurement, request to refrain from going to work when ill and report request for fever. Since COVID‐19 outbreaks are expected to occur severely in wintertime and could last until 2024,^23^ an effort is need to promote the preventive measures of COVID‐19 in the workplace during repeated outbreaks. As a randomized controlled trial reported that the adoption of the measures of body temperature each day and the obligation to stay at home for the symptomatic worker reduced the overall risk for influenza A H1N1 infection transmission by 20% in the workplace,^8^ employers can help protect workers from COVID‐19 by encouraging the use of the preventive measures of COVID‐19 and providing hazard controls to employees.^9^ Employer provision of the preventive measures of COVID‐19 in the workplace was reported to be associated with greater use of the preventive measures among all workers.^24^ Employer intentions to implement or resume these items during the COVID‐19 pandemic may be important to prevent infection in the workplace.

This study has several limitations. First, the data were collected using self‐reported questionnaires. The understanding of COVID‐19 measures might vary by individual. This study was conducted among employees but not for their companies. Therefore, the implementation rate might be underestimated. Even if companies implement measures, employees may not be aware of them. Second, the sample consisted only of full‐time employees recruited from an Internet survey company. The participants were limited to those who had access to the Internet, and they were more likely to be managers and non‐manual workers, compared to the national labor statistics in Japan.^25^ The generalizing the findings may be limited.

Third, since preventive measures in the workplace may be influenced by national policies and other factors, the generalizability of the results of this study may be limited. Fourth, participants who have changed their occupation during the survey periods were not excluded if they were working and were living in Japan. Therefore, we have not been able to take into account the effect on the results of the participants' change of occupation. Finally, the scale of workplace measures was developed through discussion among professions, and as such, it is not fully evidence‐based nor comprehensive.

## 5 CONCLUSIONS

This study reported the changes in the implementation of measures for COVID‐19 in the workplace in Japan from March to November 2020. The mean number of implemented measures for COVID‐19 in the workplace increased from March to May 2020, and did not change from May to August 2020; however, it decreased from August to November 2020. The implementation rates of various workplace COVID‐19 preventive measures, such as encourage wearing masks and enforcement of temperature measurement, significantly increased from March to May 2020, however, from August to November 2020, the implementation rates of 14 of 23 items were significantly decreased. An effort is need to encourage the workplace implementing these measures during repeated outbreaks.

## DISCLOSURE

*Approval of the research protocol:* This study was approved by the Research Ethics Committee of the Graduate School of Medicine/Faculty of Medicine, The University of Tokyo, No. 10856‐(2)(3)(4)(5). *Informed consent:* Online informed consent was obtained from all participants with full disclosure and explanation of the purpose and procedures of this study. We explained that their participation was voluntary, and they can withdraw consent for any reason, simply by not completing the questionnaire. *Registry and registration number of the study/trial:* N/A. *Animal studies:* N/A.

## CONFLICT OF INTEREST

All authors declare no relevant conflicts of interest in relation to the subject of the manuscript. NK reports grants from SB AtWork Corp, Fujitsu Ltd, and TAK Ltd, personal fees from the Occupational Health Foundation, SB AtWork Corp, RIKEN, Japan Aerospace Exploration Agency (JAXA), Japan Dental Association, Sekisui Chemicals, Junpukai Health Care Center, Osaka Chamber of Commerce and Industry, outside the submitted work.

## AUTHOR CONTRIBUTIONS

NK was in charge of this study, supervising the process and of providing his expert opinion. NS and NK organized the study design. HA, NS, and NK analyzed the data. Collaborators KI, RK, and KT ensured that questions related to the accuracy or integrity of any part of the work were appropriately investigated and resolved. HA and NK wrote the first draft of the manuscript, and all other authors critically revised it. All authors approved the final version of the manuscript.

## Data Availability

The data that support the findings of this study are available from the corresponding author upon reasonable request.
